# Does self-esteem mediate the association between perfectionism and mindfulness among Lebanese university students?

**DOI:** 10.1186/s40359-022-00964-9

**Published:** 2022-11-07

**Authors:** Emmanuelle Awad, Souheil Hallit, Sahar Obeid

**Affiliations:** 1 Social and Education Sciences Department, School of Arts and Sciences, Lebanese American University, Byblos, Lebanon; 2 School of Medicine and Medical Sciences, Holy Spirit University of Kaslik, P.O. Box 446, Jounieh, Lebanon; 3 Research Department, Psychiatric Hospital of the Cross, Jal Eddib, Lebanon

**Keywords:** Mindfulness, Perfectionism, Self-esteem, Lebanon, University students

## Abstract

**Objectives:**

To evaluate the associations between mindfulness, self-esteem and perfectionism in a Lebanese sample of university students, as well as the indirect effect of self-esteem between mindfulness and perfectionism was investigated.

**Methods:**

This cross-sectional study was carried out between July and September 2021. A total of 363 university students were recruited through convenience sampling through several universities in Lebanon’s governorates. An online survey that included a part that collected sociodemographic information, the Freiburg Mindfulness Inventory to assess mindfulness, the Rosenberg Self‐Esteem Scale to assess self-esteem and the Big Three Perfectionism Scale to assess perfectionism was completed by participants.

**Results:**

Higher self-esteem (Beta = 1.30) was significantly associated with more mindfulness, whereas higher self-critical perfectionism (Beta = − 0.61) was significantly associated with less mindfulness. Self-esteem mediated the association between self-critical and narcissistic perfectionism and mindfulness. More perfectionism was significantly and directly associated with less mindfulness and lower self-esteem, whereas higher self-esteem was significantly associated with more mindfulness.

**Conclusion:**

This study provides valuable findings that enable practitioners to effectively identify people needing interventions to improve psychological health through mindfulness, self-esteem and perfectionism. The conclusions that can be deduced from this study can help educational psychologists and counselors guide university students towards effective mindfulness practices that can increase self-esteem levels and balance maladaptive perfectionism that can cause distress and impairment in the social and academic settings.

## Background

Mindfulness is a concept that has been increasing in popularity over the last few years [1], and yet, defining it can cause some confusion to arise. From a philosophical perspective, mindfulness consists of knowing, shaping and consequently freeing the mind [2]. This tradition of wisdom is native in some Asian countries such as India, China and Japan, and has recently spread into Western societies [2]. In essence, mindfulness can be defined as an integration of attention and awareness to experience the present moment [3]. The practice of mindfulness has expanded to the field of psychology, where mindfulness-based interventions (MBIs) have been integrated and shown improvements in psychological issues such as anxiety, insomnia and stress [4]. In fact, research has shown that mindfulness has become a widely popular psychological technique to increase wellbeing, autonomy and discipline [5].

The effect of mindfulness-based interventions has extended to other psychological variables, including self-esteem. A multitude of studies has found a positive association between MBIs and self-esteem, where adopting mindfulness has significantly increased self-esteem [6]. Additionally, mindfulness training improved positive self-esteem and predicted it in another study [7]. This was evident in a sample of university students, where practicing mindfulness produced improvements in both self-esteem and growth mindset in the educational and social settings [8]. In addition, the practice of mindfulness has contributed to the stability of self-esteem over time [9]. Self-esteem also influenced mindfulness, as it mediated its relationship with happiness among university students [10]. Similarly, self-esteem mediated the association between mindfulness and psychological wellbeing [11]. Previous literature suggests that self-esteem has an indirect effect on mindfulness, such as the case in another recent study where self-esteem was a pathway for a positive relationship between mindfulness and life satisfaction [12].

Apart from self-esteem, mindfulness in combination with adaptive perfectionism was found to have positive effects on both physical and psychological wellbeing [13]. However, perfectionism is not always adaptive or beneficial psychologically. In fact, mindfulness-based therapy was used in hand with cognitive therapy to cope with distress related to perfectionism [14]. The use of such mindfulness practices was helpful for a sample of university students in dealing with impairment due to perfectionism [14]. The lack of mindfulness practices was associated with maladaptive perfectionism that negatively influenced the ability to deal with life’s difficulties [15]. Similarly, university students who encounter increased pressure to be perfect were found to benefit from practicing mindfulness in recent studies [16]. Here, it is important to differentiate between the three perfectionism dimensions that are discussed in the present study. To assess perfectionism, the Big Three Perfectionism Scale was adopted, with three factors: rigid perfectionism, which consists of insisting on having flawless, perfect and error-free performance, a strong necessity to be perfect and flawless [17]; self-critical perfectionism, which is characterized by scrutinizing one’s mistakes, self-doubt and self-criticism, worry about having imperfect performance and experiencing distress if performance is perceived as error-prone [17]; and narcissistic perfectionism, which is directed towards others through grandiose behavior, entitlement and over-criticism of others, demanding others to be perfect and experiencing feelings of distress if others’ performance is perceived as imperfect [17, 18]. Given that rigid and self-critical perfectionism are directed towards the self, it has been noted to have the most prominent maladaptive qualities [19], as opposed to narcissistic perfectionism. Generally, maladaptive perfectionism is associated with lower self-esteem [20]. In another study, adaptive perfectionism that represented personal striving was related to higher self-esteem while self-esteem associated with criticism was related to lower self-esteem [21]. Another study found a negative relationship between self-esteem and maladaptive perfectionism among students [22].

In a study that included a Lebanese sample of university students, results showed that people with higher self-esteem and lower perfectionism scores were less likely to exhibit maladaptive behavior such as justifying failure and internalizing invalid success [23]. Another study in Lebanon showed that self-compassion, which can be considered an antonym to maladaptive perfectionism, and mindfulness were positively and significantly associated with each other in a sample of middle school students [24]. As previously mentioned, rigid and self-critical perfectionism involve stressful mechanisms that pressure the self, and negative perfectionism is related to lower mindfulness. These supported assumptions can be backed-up by a study involving Lebanese adolescents, where stress was more likely to be lower if mindfulness is practiced [25]. Furthermore, self-esteem mediated the relationship between mindfulness and other psychological variables in multiple studies. In a sample of Chinese university students, self-esteem mediated the association between mindfulness and social anxiety [26]. Perfectionism is connected to social anxiety in a core element: criticism and the individual believing that he/she is not meeting certain expectations [27]. Similarly, self-esteem was a full mediator in the association between mindfulness and psychological wellbeing [28].

Through research, it can be inferred that the studies investigating mindfulness, self-esteem and perfectionism are scarce, rather non-existent for the Lebanese population. The topic of mindfulness has not been scientifically explored within Lebanon yet. It is an understatement to say that the devastating economic and social tragedies that have been occurring in Lebanon affect the state of university students. Furthermore, previous studies have explored the relationships involved with self-esteem such as stress [29] and depression [30] among university students. However, none investigated the associations between self-esteem, mindfulness and perfectionism within university students, to the best of our knowledge.

A previous study found that about 75% of Lebanese university students developed acute stress [31]. Meanwhile, mindfulness was a significant influencer on bettering university students’ psychological health [32]. Therefore, examining mindfulness and its associated psychological variables among university students is most valuable and beneficial for interventions at a time that can be described as the worst period in modern Lebanese history. Hence, in this study, the aim was to evaluate the associations between mindfulness, self-esteem and perfectionism in a Lebanese sample of university students. Additionally, the indirect effect of self-esteem between mindfulness and perfectionism was investigated. The current study is directed towards healthcare professionals and scientists, especially those who work with university students, a population that faces academic, social and country-related pressures, in order to provide more information about the variables at hand and guide action.

## Methods

### Study design and participants

This cross-sectional study was carried out between July and September 2021. A total of 363 university students were recruited through convenience sampling through several universities in Lebanon’s governorates. Participants received the online link to the survey. Involved people were encouraged to visit a website that would guide them to the consent form, information form (purpose of the current study, anonymity, voluntariness of consent to research), and questionnaire. All participants responded willingly to the survey. There were no fees for participating in the study. All university students over the age of 18 were eligible to participate. Excluded were those who refused to complete the survey [33, 34].

### Minimal sample size calculation

According to the G-power, a minimum of 316 students was deemed necessary to have enough statistical power, based on a 5% risk of error, 80% power, f^2^ = 2.5% and 10 factors to be entered in the multivariable analysis.

### Questionnaire and variables

The Arabic self-administered questionnaire with closed-ended questions was anonymous; the questionnaire required approximately 20 min to be completed. The questionnaire consisted of different sections. The first part clarified socio-demographic characteristics: age, gender, marital status, and household crowding index. The latter, reflecting the socioeconomic status of the family, was calculated by dividing the number of persons in the house by the number of rooms in the house excluding the bathrooms and kitchen [35]. The physical activity index was calculated by multiplying the intensity by the frequency by the time of physical activity [36].

The second part of the questionnaire included the following scales:

### Freiburg mindfulness inventory (FMI)

Freiburg Mindfulness Inventory (FMI) is composed of 14 items describing all aspects of mindfulness [37]. This instrument is used to characterize the person’s experience of mindfulness. Each item is scored based on a 4-point Likert scale with 1 = rarely and 4 = always. Higher total score means more mindfulness. This scale is validated in Lebanon [38]. The Cronbach’s alpha in this study was 0.92.

### Rosenberg self‐esteem scale (RSES)

It is a 10-item scale that reflects self-worth by focusing on both positive and negative feelings people have about themselves [39]. Items are scored on a four-point Likert scale (1 = strongly disagree to 4 = strongly agree). Higher scores reflect a better self-esteem. The Arabic version has been used in previous papers [40, 41]. The Cronbach’s alpha in this study was 0.99.

### Big three perfectionism scale

This scale is composed of 16 items, scored on a five-point Likert scale (1 = strongly disagree to 5 = strongly agree) [18]. It yields three subscales scores: rigid perfectionism, self-critical perfectionism and narcissistic perfectionism. Higher scores reflect higher perfectionism in the three aspects. In this study, the Cronbach’s alpha values for the three scores were as follows: rigid perfectionism (α = 0.87), self-critical perfectionism (α = 0.88) and narcissistic perfectionism (α = 0.81).

### Statistical analysis

SPSS software version 25 was used to conduct data analysis. The normality of the mindfulness, self-esteem and perfectionism subscales scores were verified via the skewness and kurtosis values varying between − 1 and + 1 [42]. A bivariate analysis using the Pearson correlation test served to assess the relationship between the mindfulness score and other continuous variables, whereas the Student t test was used to compare two means. A linear regression was conducted taking mindfulness as the dependent variable. The PROCESS SPSS Macro v. 3.4, Model 4 [43] was used to conduct the mediation analysis; three pathways were calculated: (a) Relation between perfectionism and SE; (b) Relation between SE and mindfulness; (c’) Direct effect of the relation between perfectionism and mindfulness. Pathway AB determined the indirect effect; the latter was considered significant if the confidence interval did not pass by zero. The mediation analysis and linear regression results were adjusted over all variables that showed a *p* < 0.25 in the bivariate analysis for the elimination of confounding factors as much as possible. Significance was defined at *p* < 0.05.

## Results

### Sociodemographic and other characteristics of the participants

The mean age of the participants was 22.65 ± 3.48 years, with 61.7% females. Other characteristics are summarized in Table 1.

**Table 1:** Sociodemographic and other characteristics of the participants (*N* = 363)

Variable	*N* (%)
*Sex*	
Male	139 (38.3%)
Female	224 (61.7%)
*Marital status*	
Single	343 (94.5%)
Married	20 (5.5%)

	Mean ± SD
Age (in years)	22.65 ± 3.48
Physical activity index	27.94 ± 20.44
Household crowding index	1.01 ± 0.53
Self-esteem	16.14 ± 2.09
Rigid perfectionism	12.61 ± 3.80
Self-critical perfectionism	17.60 ± 5.63
Narcissistic perfectionism	14.29 ± 4.76
Mindfulness	24.32 ± 8.56

### Bivariate analysis

A higher mean mindfulness score was found in married participants compared to single ones (30.20 vs. 23.98; *p* = 0.001) (Table 2). Older age (r = 0.15), and higher self-esteem (r = 0.43) were significantly associated with more mindfulness, whereas higher rigid (r = − 0.15), self-critical (r = − 0.45) and narcissistic (r = − 0.19) perfectionism were significantly associated with less mindfulness (Table 3).

**Table 2:** Bivariate analysis of the categorical variables associated with mindfulness

Variable	Mindfulness	
	Mean ± SD	*p*
*Sex*		0.390
Male	24.81 ± 8.09	
Female	24.02 ± 8.84	
*Marital status*		**0.001**
Single	23.98 ± 8.53	
Married	30.20 ± 6.97	

Numbers in bold indicate significant *p*-values

**Table 3:** Correlation of continuous variables with mindfulness

Variable	Mindfulness	
	r	*p*
Age	0.15	**0.004**
Physical activity index	0.03	0.539
Household crowding index	− 0.08	0.126
Rigid perfectionism	− 0.15	**0.006**
Self-critical perfectionism	− 0.45	** < 0.001**
Narcissistic perfectionism	− 0.19	** < 0.001**
Self-esteem	0.43	** < 0.001**

Numbers in bold indicate significant *p*-values; r = Pearson correlation coefficient

### Multivariable analysis

A linear regression taking mindfulness as the dependent variable, showed that higher self-esteem (Beta = 1.30) was significantly associated with more mindfulness, whereas higher self-critical perfectionism (Beta = − 0.61) was significantly associated with less mindfulness (Table 4).

**Table 4:** Multivariable analysis: Linear regression (using the ENTER method) taking mindfulness as the dependent variable (Nagelkerke R^2^ = 31.9%)

	Beta	β	*p*	95% CI
Marital status (married vs. single*)	1.93	0.05	0.345	− 2.08; 5.93
Age	0.21	0.09	0.110	− 0.05; 0.48
Household crowding index	− 0.17	− 0.01	0.810	− 1.59; 1.24
Self-esteem	1.30	0.32	** < 0.001**	0.92; 1.68
Rigid perfectionism	0.12	0.05	0.332	− 0.12; 0.37
Self-critical perfectionism	− 0.61	− 0.40	** < 0.001**	− 0.78; − 0.43
Narcissistic perfectionism	0.11	0.06	0.274	− 0.08; 0.30

*Reference group; Beta = unstandardized beta; β = standardized beta; CI = Confidence interval; numbers in bold indicate significant *p*-values

### Mediation analysis

**Fig. 1:**
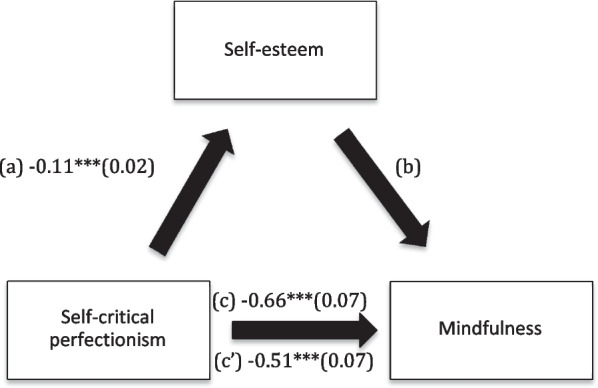
**a** Relation between self-critical perfectionism and self-esteem (R^2^ = 9.88%); **b** Relation between self-esteem and mindfulness (R^2^ = 31.36%); **c** Total effect between self-critical perfectionism and mindfulness; **c’** Direct effect between self-critical perfectionism and mindfulness (R^2^ = 22.10%). Numbers are displayed as regression coefficients (standard error). ***p < 0.001

**Fig. 2:**
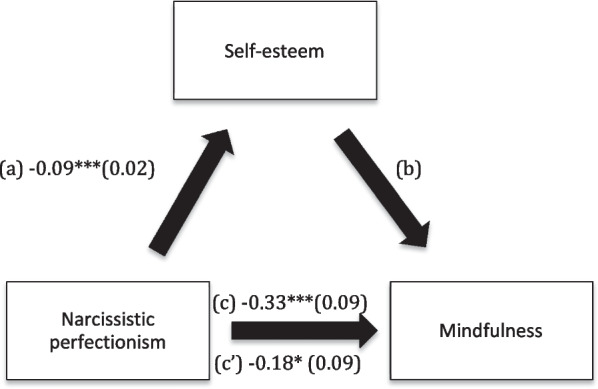
**a** Relation between narcissistic perfectionism and self-esteem (R^2^ = 5.18%); **b** Relation between self-esteem and mindfulness (R^2^ = 22.11%); **c** Total effect between narcissistic perfectionism and mindfulness; **c’** Direct effect between narcissistic perfectionism and mindfulness (R^2^ = 6.87%). Numbers are displayed as regression coefficients (standard error). ***p < 0.001, *p < 0.05

The results of the mediation analysis showed that self-esteem mediated the association between self-critical and narcissistic perfectionism and mindfulness (Table 5). More perfectionism was significantly and directly associated with less mindfulness and lower self-esteem, whereas higher self-esteem was significantly associated with more mindfulness (Figs. 1 and 2).

**Table 5:** Mediation analyses results, taking perfectionism as the independent variable, self-esteem as the mediator and mindfulness as the dependent variable

	Direct effect			Indirect effect		
	Beta	SE	*P*	Beta	Boot SE	Boot CI
Rigid perfectionism	− 0.29	0.11	0.006	− 0.04	0.05	− 0.15; 0.06
Self-critical perfectionism	− 0.51	0.07	< 0.001	− 0.15	0.03	− 0.21; − 0.09*
Narcissistic perfectionism	− 0.18	0.09	0.036	− 0.15	0.04	− 0.23; − 0.07*

* indicates significant mediation

## Discussion

### Mindfulness, self-esteem and perfectionism

Higher self-esteem was significantly associated with more mindfulness. An older study showed that individuals with higher and stable self-esteem levels were more likely to practice mindfulness [44]. Another investigation showed that enhancing self-esteem amplified the positive effects of mindfulness, which subsequently reflected positively on other behavioral problems [45]. Having said that, the majority of studies evaluating the relationship between self-esteem and mindfulness examine the effect of mindfulness on self-esteem, or determine the presence of a significant correlation. For example, a recent study that involved an Indian sample of adults, where mindfulness techniques reflected positively on the level of self-esteem [46]. Another study hypothesized that mindfulness contributes to better psychological wellbeing, which increases the probability of having a higher self-esteem [47]. Similar results were shown in a sample of university students as mindfulness practices significantly increased the level of self-esteem, even when the mindfulness practice was brief [7]. Also, mindfulness was able to buffer the adverse effects of low self-esteem among a sample of university students [48]. These results combined can indicate the positive correlation between self-esteem and mindfulness, further implying that higher self-esteem and higher mindfulness are related.

Higher self-critical perfectionism was significantly associated with less mindfulness. Research has shown that individuals with high self-critical perfectionism experience more distress and hassles in comparison with other university students [49]. Practicing mindfulness and distress have an inverse relationship: higher mindfulness is associated with lower distress [50]. Therefore, it might be inferred that the negative association between self-critical perfectionism and mindfulness is rational. In a recent study, self-critical perfectionism not only was associated with less mindfulness, but also predicted higher distress within the next two years [51]. Mindfulness is more likely to reduce self-critique and judgmental attitudes towards the self [47].

More perfectionism was significantly and directly associated with less mindfulness and lower self-esteem, whereas higher self-esteem was significantly associated with more mindfulness. An older study set the foundation for these findings: people who had perfectionist self-standards and met the criteria for perfectionism in a study had lower self-esteem [52]. Later, it was found that individuals exhibiting higher maladaptive perfectionism were more likely to have lower self-esteem [20]. These results were supported by another study that showed a significant association between perfectionism and decreased levels of self-esteem among university students [53]. In a previous study, a negative correlation was found between perfectionism and psychological wellbeing while a positive relationship was found between self-esteem and psychological wellbeing [54]. As for the association between perfectionism and mindfulness, a study showed that perfectionism is significantly related to low mindfulness [16]. This relationship can be supported by a recent study where higher perfectionism levels were associated with lower mindfulness [55]. Given these points discussed above, it can be hypothesized that individuals with high perfectionism levels are more likely to have low self-esteem and subsequently less likely to practice mindfulness.

### Self-esteem mediation between mindfulness and perfectionism

Self-esteem mediated the association between self-critical and narcissistic perfectionism, and mindfulness. Previous studies have shown that self-esteem mediated the relationship between perfectionism and other psychological variables such as self-efficacy [56]. Another study showed that self-esteem mediated the association between maladaptive perfectionism and depression among a sample of university students [57]. Results also indicated that self-esteem was a full mediator between mindfulness and psychological wellbeing for a sample of undergraduate university students [28]. Additionally, self-esteem mediated the connection between self-critical perfectionism and depressive symptoms [58]. It can be observed from the results stated that no studies have investigated the mediation role of self-esteem on the relationship between perfectionism and mindfulness. It is important to note that past investigation found that both self-critical and narcissistic perfectionism dimensions are associated with lower self-esteem among university students [53]. In addition, self-critical perfectionism is related to lower mindfulness [51] while there is a lack of studies on the association between mindfulness and narcissistic perfectionism. It can be hypothesized that higher mindfulness increases the probability of having higher self-esteem, which in turn affects the manifestation of perfectionism in either the self-critical or narcissistic dimension.

### Mindfulness practices, self-esteem and perfectionism

Multiple studies have aimed to investigate the efficacy of mindfulness practices on mental health variables. Mindfulness-based cognitive therapy (MBCT) is a practice used to raise mindfulness, aid in perceiving experiences in a neutral way and avoid negative thought processes [59]. Research indicates that attending MBCT effectively improved self-esteem levels in a previous study [60]. Other analyses showed that MBCT aids in improving multiple psychological variables and prevents the recurrence of psychiatric disorders [61]. Additionally, MBCT was found to significantly decrease levels of perfectionism in an intervention program lasting 8 weeks [14]. Another experiment also showed a significant improvement on self-esteem measures following MBCT training sessions [62]. These findings further highlight the importance of the associations that were established in our study.

### Clinical implications

The results of the current study highlight the importance of mindfulness and its practices on improving psychological variables such as self-esteem and perfectionism. These findings are especially important as the study was conducted on university students, a sample that is under substantial pressure to perform socially and academically. The conclusions that can be deduced from this study can help educational psychologists and counselors guide university students towards effective mindfulness practices that can increase self-esteem levels and balance maladaptive perfectionism that can cause distress and impairment in the social and academic settings.

### Limitations

First, the study was executed on a sample of university students, which means that the results cannot be generalized to the entire Lebanese population. Second, the analyses conducted reflect correlational relationships between the variables discussed and therefore do not infer causation. For future research, it is recommended to replicate this study longitudinally. Third, self‐report measures were used to assess the different study variables, which could be subject to social desirability bias. Fourth, not all scales used in this research have been validated in Lebanon yet (i.e. The Big three perfectionism scale). Additionally, other factors (year of study, major, type of university, type of faculty), which might be related to mental health, have not been assessed in the questionnaire, predisposing us to a confounding bias.

## Conclusion

This study provides valuable findings that enable practitioners to effectively identify people needing interventions to improve psychological health through mindfulness, self-esteem and perfectionism. The current results are novel within the Lebanese context and add new information to a gap in the existing body of literature about self-esteem, mindfulness and perfectionism. Having established significant associations between the variables at hand, future studies are needed to explore specific mindfulness practices that are most strongly in relation to self-esteem and the different dimensions of perfectionism. This could help in the employment of specific mindfulness practices towards the benefit of people’s psychological health. Furthermore, longitudinal studies are needed to determine the longevity of the effect of mindfulness in its different practices on the Lebanese population, as mindfulness practices could submit to cultural variations.

## Data Availability

All data generated or analyzed during this study are not publicly available due the restrictions from the ethics committee. Any request about the data can be sent to the corresponding author (SH).
